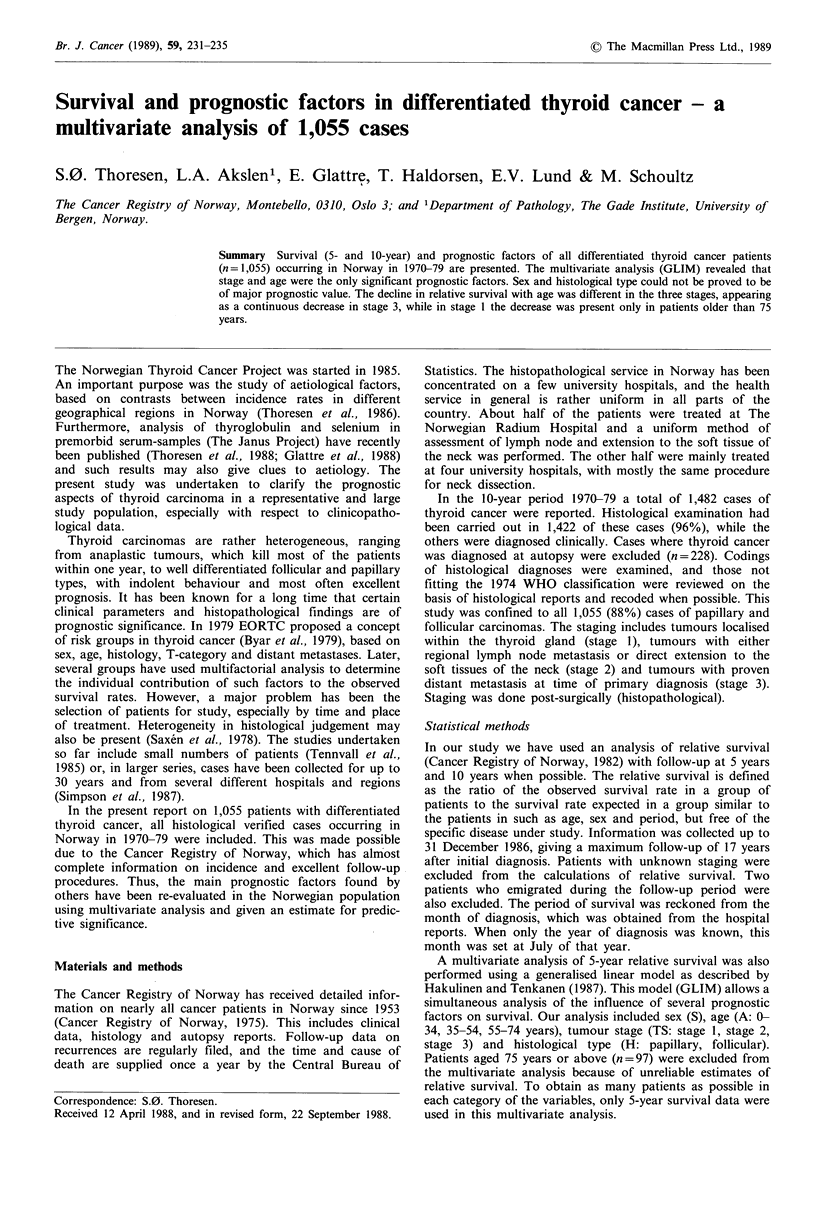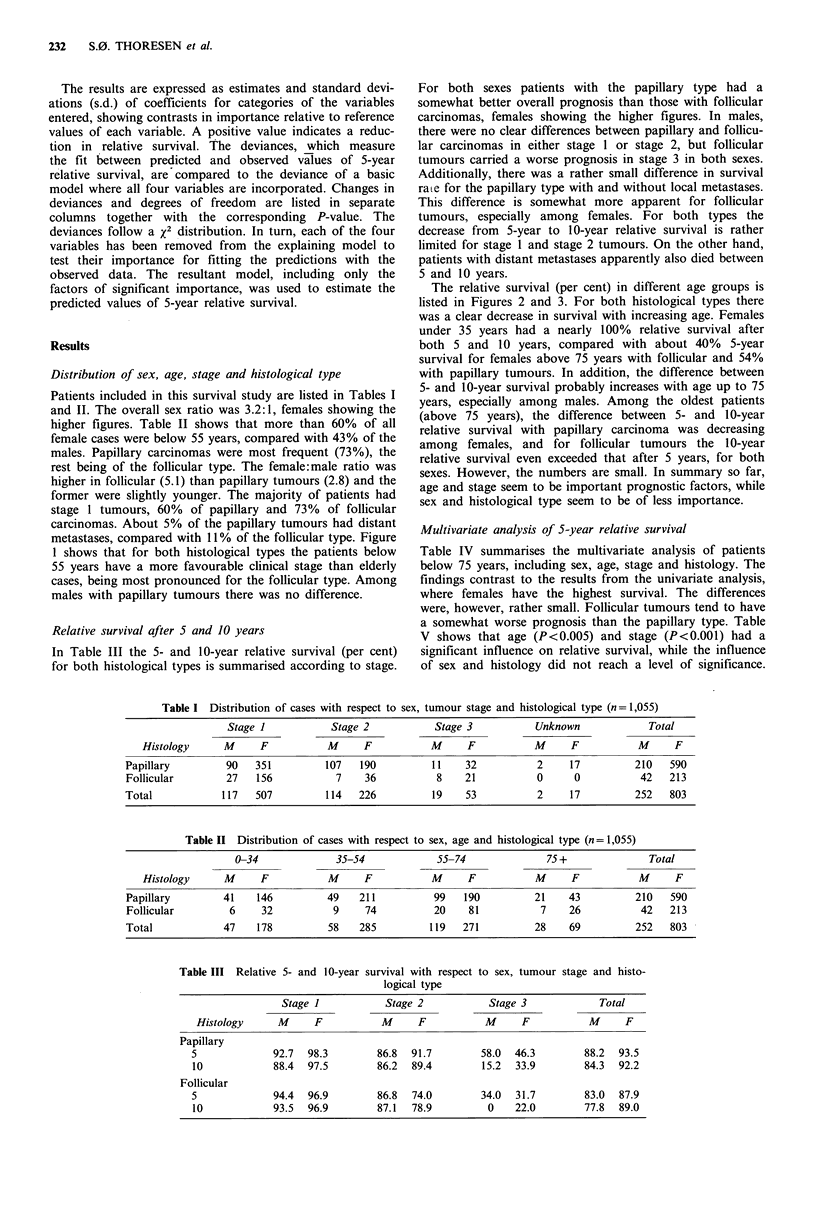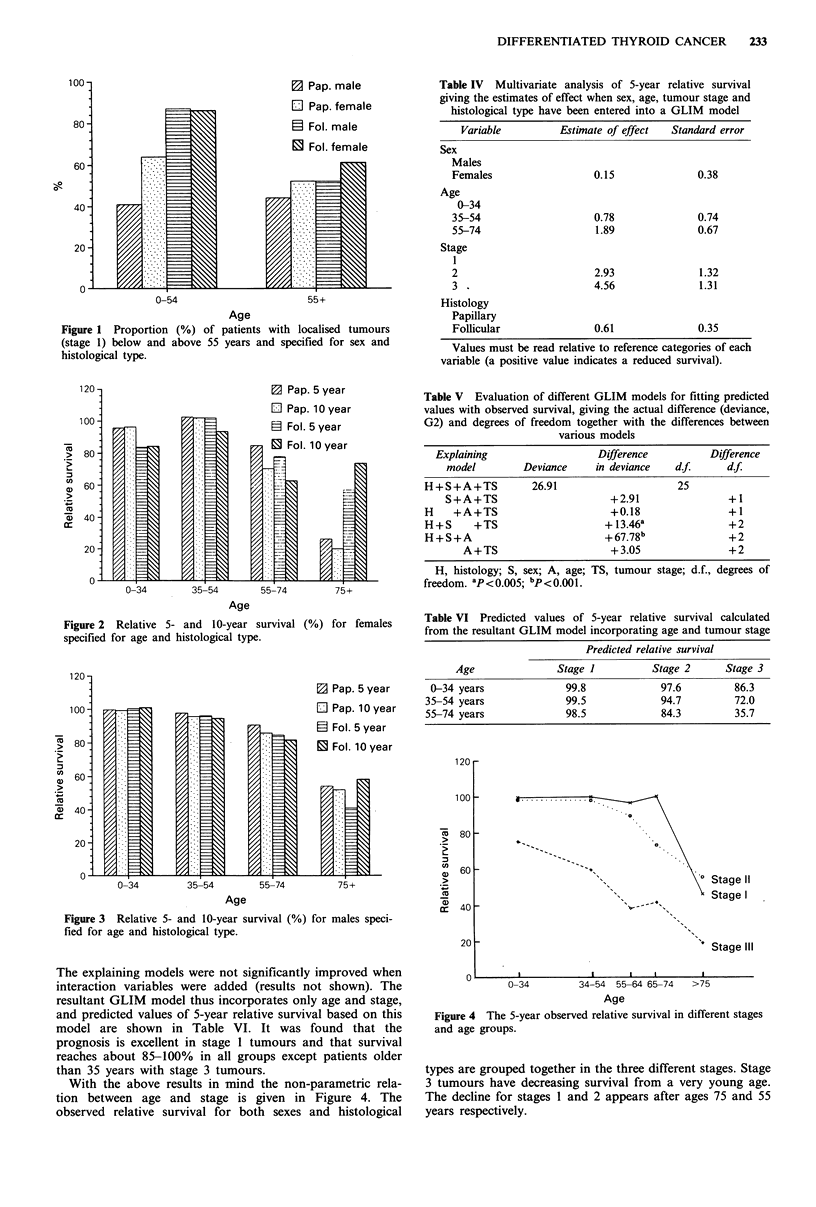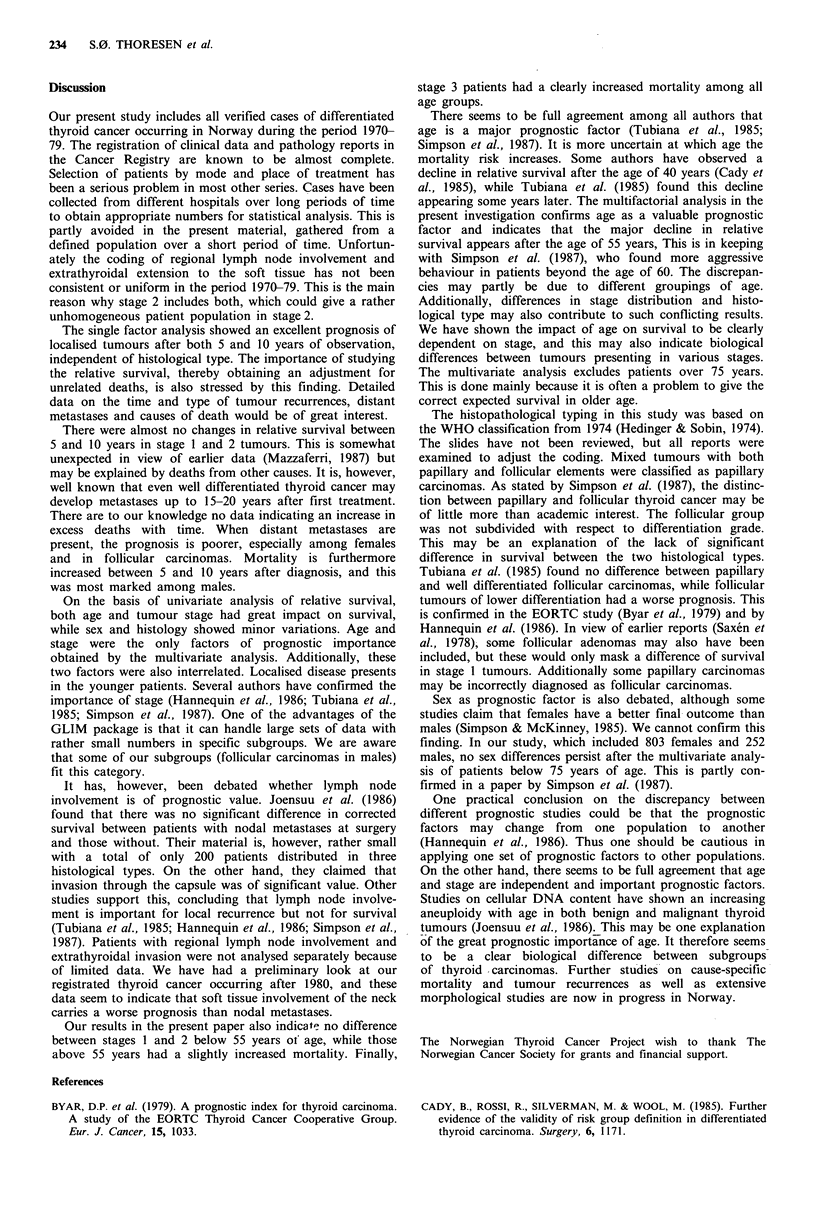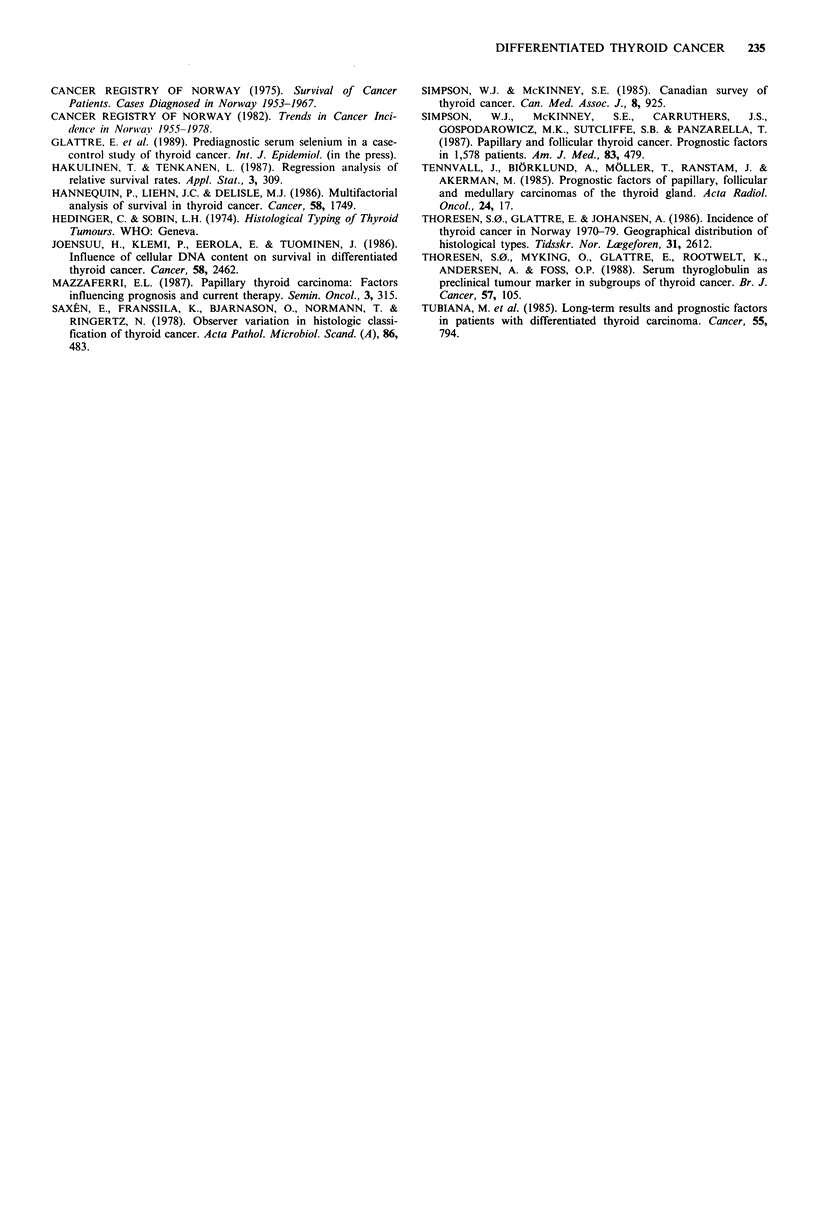# Survival and prognostic factors in differentiated thyroid cancer--a multivariate analysis of 1,055 cases.

**DOI:** 10.1038/bjc.1989.47

**Published:** 1989-02

**Authors:** S. O. Thoresen, L. A. Akslen, E. Glattre, T. Haldorsen, E. V. Lund, M. Schoultz

**Affiliations:** Cancer Registry of Norway, Oslo.

## Abstract

Survival (5- and 10-year) and prognostic factors of all differentiated thyroid cancer patients (n = 1,055) occurring in Norway in 1970-79 are presented. The multivariate analysis (GLIM) revealed that stage and age were the only significant prognostic factors. Sex and histological type could not be proved to be of major prognostic value. The decline in relative survival with age was different in the three stages, appearing as a continuous decrease in stage 3, while in stage 1 the decrease was present only in patients older than 75 years.


*Br. J. Cancer* (1989), **59**, 231–235 © The Macmillan Press Ltd., 1989

### Survival and prognostic factors in differentiated thyroid cancer – a multivariate analysis of 1,055 cases

S.Ø. Thoresen, L.A. Akslen[1], E. Glattre, T. Haldorsen, E.V. Lund & M. Schoultz

*The Cancer Registry of Norway, Montebello, 0310, Oslo 3; and [1]Department of Pathology, The Gade Institute, University of Bergen, Norway.*

**Summary** Survival (5- and 10-year) and prognostic factors of all differentiated thyroid cancer patients ($n=1{,}055$) occurring in Norway in 1970–79 are presented. The multivariate analysis (GLIM) revealed that stage and age were the only significant prognostic factors. Sex and histological type could not be proved to be of major prognostic value. The decline in relative survival with age was different in the three stages, appearing as a continuous decrease in stage 3, while in stage 1 the decrease was present only in patients older than 75 years.

The Norwegian Thyroid Cancer Project was started in 1985. An important purpose was the study of aetiological factors, based on contrasts between incidence rates in different geographical regions in Norway (Thoresen *et al.*, 1986). Furthermore, analysis of thyroglobulin and selenium in premorbid serum-samples (The Janus Project) have recently been published (Thoresen *et al.*, 1988; Glattre *et al.*, 1988) and such results may also give clues to aetiology. The present study was undertaken to clarify the prognostic aspects of thyroid carcinoma in a representative and large study population, especially with respect to clinicopathological data.

Thyroid carcinomas are rather heterogeneous, ranging from anaplastic tumours, which kill most of the patients within one year, to well differentiated follicular and papillary types, with indolent behaviour and most often excellent prognosis. It has been known for a long time that certain clinical parameters and histopathological findings are of prognostic significance. In 1979 EORTC proposed a concept of risk groups in thyroid cancer (Byar *et al.*, 1979), based on sex, age, histology, T-category and distant metastases. Later, several groups have used multifactorial analysis to determine the individual contribution of such factors to the observed survival rates. However, a major problem has been the selection of patients for study, especially by time and place of treatment. Heterogeneity in histological judgement may also be present (Saxèn *et al.*, 1978). The studies undertaken so far include small numbers of patients (Tennvall *et al.*, 1985) or, in larger series, cases have been collected for up to 30 years and from several different hospitals and regions (Simpson *et al.*, 1987).

In the present report on 1,055 patients with differentiated thyroid cancer, all histological verified cases occurring in Norway in 1970–79 were included. This was made possible due to the Cancer Registry of Norway, which has almost complete information on incidence and excellent follow-up procedures. Thus, the main prognostic factors found by others have been re-evaluated in the Norwegian population using multivariate analysis and given an estimate for predictive significance.

### Materials and methods

The Cancer Registry of Norway has received detailed information on nearly all cancer patients in Norway since 1953 (Cancer Registry of Norway, 1975). This includes clinical data, histology and autopsy reports. Follow-up data on recurrences are regularly filed, and the time and cause of death are supplied once a year by the Central Bureau of Statistics. The histopathological service in Norway has been concentrated on a few university hospitals, and the health service in general is rather uniform in all parts of the country. About half of the patients were treated at The Norwegian Radium Hospital and a uniform method of assessment of lymph node and extension to the soft tissue of the neck was performed. The other half were mainly treated at four university hospitals, with mostly the same procedure for neck dissection.

In the 10-year period 1970–79 a total of 1,482 cases of thyroid cancer were reported. Histological examination had been carried out in 1,422 of these cases (96%), while the others were diagnosed clinically. Cases where thyroid cancer was diagnosed at autopsy were excluded ($n=228$). Codings of histological diagnoses were examined, and those not fitting the 1974 WHO classification were reviewed on the basis of histological reports and recoded when possible. This study was confined to all 1,055 (88%) cases of papillary and follicular carcinomas. The staging includes tumours localised within the thyroid gland (stage 1), tumours with either regional lymph node metastasis or direct extension to the soft tissues of the neck (stage 2) and tumours with proven distant metastasis at time of primary diagnosis (stage 3). Staging was done post-surgically (histopathological).

### *Statistical methods*

In our study we have used an analysis of relative survival (Cancer Registry of Norway, 1982) with follow-up at 5 years and 10 years when possible. The relative survival is defined as the ratio of the observed survival rate in a group of patients to the survival rate expected in a group similar to the patients in such as age, sex and period, but free of the specific disease under study. Information was collected up to 31 December 1986, giving a maximum follow-up of 17 years after initial diagnosis. Patients with unknown staging were excluded from the calculations of relative survival. Two patients who emigrated during the follow-up period were also excluded. The period of survival was reckoned from the month of diagnosis, which was obtained from the hospital reports. When only the year of diagnosis was known, this month was set at July of that year.

A multivariate analysis of 5-year relative survival was also performed using a generalised linear model as described by Hakulinen and Tenkanen (1987). This model (GLIM) allows a simultaneous analysis of the influence of several prognostic factors on survival. Our analysis included sex (S), age (A: 0–34, 35–54, 55–74 years), tumour stage (TS: stage 1, stage 2, stage 3) and histological type (H: papillary, follicular). Patients aged 75 years or above ($n=97$) were excluded from the multivariate analysis because of unreliable estimates of relative survival. To obtain as many patients as possible in each category of the variables, only 5-year survival data were used in this multivariate analysis.

Correspondence: S.Ø. Thoresen.
Received 12 April 1988, and in revised form, 22 September 1988.

The results are expressed as estimates and standard deviations (s.d.) of coefficients for categories of the variables entered, showing contrasts in importance relative to reference values of each variable. A positive value indicates a reduction in relative survival. The deviances, which measure the fit between predicted and observed values of 5-year relative survival, are compared to the deviance of a basic model where all four variables are incorporated. Changes in deviances and degrees of freedom are listed in separate columns together with the corresponding $P$-value. The deviances follow a $\chi^2$ distribution. In turn, each of the four variables has been removed from the explaining model to test their importance for fitting the predictions with the observed data. The resultant model, including only the factors of significant importance, was used to estimate the predicted values of 5-year relative survival.

### Results

### *Distribution of sex, age, stage and histological type*

Patients included in this survival study are listed in Tables I and II. The overall sex ratio was 3.2:1, females showing the higher figures. Table II shows that more than 60% of all female cases were below 55 years, compared with 43% of the males. Papillary carcinomas were most frequent (73%), the rest being of the follicular type. The female:male ratio was higher in follicular (5.1) than papillary tumours (2.8) and the former were slightly younger. The majority of patients had stage 1 tumours, 60% of papillary and 73% of follicular carcinomas. About 5% of the papillary tumours had distant metastases, compared with 11% of the follicular type. Figure 1 shows that for both histological types the patients below 55 years have a more favourable clinical stage than elderly cases, being most pronounced for the follicular type. Among males with papillary tumours there was no difference.

### *Relative survival after 5 and 10 years*

In Table III the 5- and 10-year relative survival (per cent) for both histological types is summarised according to stage. For both sexes patients with the papillary type had a somewhat better overall prognosis than those with follicular carcinomas, females showing the higher figures. In males, there were no clear differences between papillary and follicular carcinomas in either stage 1 or stage 2, but follicular tumours carried a worse prognosis in stage 3 in both sexes. Additionally, there was a rather small difference in survival raıe for the papillary type with and without local metastases. This difference is somewhat more apparent for follicular tumours, especially among females. For both types the decrease from 5-year to 10-year relative survival is rather limited for stage 1 and stage 2 tumours. On the other hand, patients with distant metastases apparently also died between 5 and 10 years.

The relative survival (per cent) in different age groups is listed in Figures 2 and 3. For both histological types there was a clear decrease in survival with increasing age. Females under 35 years had a nearly 100% relative survival after both 5 and 10 years, compared with about 40% 5-year survival for females above 75 years with follicular and 54% with papillary tumours. In addition, the difference between 5- and 10-year survival probably increases with age up to 75 years, especially among males. Among the oldest patients (above 75 years), the difference between 5- and 10-year relative survival with papillary carcinoma was decreasing among females, and for follicular tumours the 10-year relative survival even exceeded that after 5 years, for both sexes. However, the numbers are small. In summary so far, age and stage seem to be important prognostic factors, while sex and histological type seem to be of less importance.

### *Multivariate analysis of 5-year relative survival*

Table IV summarises the multivariate analysis of patients below 75 years, including sex, age, stage and histology. The findings contrast to the results from the univariate analysis, where females have the highest survival. The differences were, however, rather small. Follicular tumours tend to have a somewhat worse prognosis than the papillary type. Table V shows that age ($P<0.005$) and stage ($P<0.001$) had a significant influence on relative survival, while the influence of sex and histology did not reach a level of significance.

**Table I** Distribution of cases with respect to sex, tumour stage and histological type ($n=1,055$)

| | *Stage 1* | | *Stage 2* | | *Stage 3* | | *Unknown* | | *Total* | |
|---|---|---|---|---|---|---|---|---|---|---|
| *Histology* | *M* | *F* | *M* | *F* | *M* | *F* | *M* | *F* | *M* | *F* |
| Papillary | 90 | 351 | 107 | 190 | 11 | 32 | 2 | 17 | 210 | 590 |
| Follicular | 27 | 156 | 7 | 36 | 8 | 21 | 0 | 0 | 42 | 213 |
| Total | 117 | 507 | 114 | 226 | 19 | 53 | 2 | 17 | 252 | 803 |

**Table II** Distribution of cases with respect to sex, age and histological type ($n=1,055$)

| | *0–34* | | *35–54* | | *55–74* | | *75+* | | *Total* | |
|---|---|---|---|---|---|---|---|---|---|---|
| *Histology* | *M* | *F* | *M* | *F* | *M* | *F* | *M* | *F* | *M* | *F* |
| Papillary | 41 | 146 | 49 | 211 | 99 | 190 | 21 | 43 | 210 | 590 |
| Follicular | 6 | 32 | 9 | 74 | 20 | 81 | 7 | 26 | 42 | 213 |
| Total | 47 | 178 | 58 | 285 | 119 | 271 | 28 | 69 | 252 | 803 |

**Table III** Relative 5- and 10-year survival with respect to sex, tumour stage and histological type

| | *Stage 1* | | *Stage 2* | | *Stage 3* | | *Total* | |
|---|---|---|---|---|---|---|---|---|
| *Histology* | *M* | *F* | *M* | *F* | *M* | *F* | *M* | *F* |
| Papillary | | | | | | | | |
| 5 | 92.7 | 98.3 | 86.8 | 91.7 | 58.0 | 46.3 | 88.2 | 93.5 |
| 10 | 88.4 | 97.5 | 86.2 | 89.4 | 15.2 | 33.9 | 84.3 | 92.2 |
| Follicular | | | | | | | | |
| 5 | 94.4 | 96.9 | 86.8 | 74.0 | 34.0 | 31.7 | 83.0 | 87.9 |
| 10 | 93.5 | 96.9 | 87.1 | 78.9 | 0 | 22.0 | 77.8 | 89.0 |

**Figure 1** Proportion (%) of patients with localised tumours (stage 1) below and above 55 years and specified for sex and histological type.

**Figure 2** Relative 5- and 10-year survival (%) for females specified for age and histological type.

**Figure 3** Relative 5- and 10-year survival (%) for males specified for age and histological type.

The explaining models were not significantly improved when interaction variables were added (results not shown). The resultant GLIM model thus incorporates only age and stage, and predicted values of 5-year relative survival based on this model are shown in Table VI. It was found that the prognosis is excellent in stage 1 tumours and that survival reaches about 85–100% in all groups except patients older than 35 years with stage 3 tumours.

With the above results in mind the non-parametric relation between age and stage is given in Figure 4. The observed relative survival for both sexes and histological types are grouped together in the three different stages. Stage 3 tumours have decreasing survival from a very young age. The decline for stages 1 and 2 appears after ages 75 and 55 years respectively.

**Table IV** Multivariate analysis of 5-year relative survival giving the estimates of effect when sex, age, tumour stage and histological type have been entered into a GLIM model

| *Variable* | *Estimate of effect* | *Standard error* |
|---|---|---|
| Sex | | |
| Males | | |
| Females | 0.15 | 0.38 |
| Age | | |
| 0–34 | | |
| 35–54 | 0.78 | 0.74 |
| 55–74 | 1.89 | 0.67 |
| Stage | | |
| 1 | | |
| 2 | 2.93 | 1.32 |
| 3 . | 4.56 | 1.31 |
| Histology | | |
| Papillary | | |
| Follicular | 0.61 | 0.35 |

Values must be read relative to reference categories of each variable (a positive value indicates a reduced survival).

**Table V** Evaluation of different GLIM models for fitting predicted values with observed survival, giving the actual difference (deviance, G2) and degrees of freedom together with the differences between various models

| *Explaining model* | *Deviance* | *Difference in deviance* | *d.f.* | *Difference d.f.* |
|---|---|---|---|---|
| H+S+A+TS | 26.91 | | 25 | |
| S+A+TS | | +2.91 | | +1 |
| H +A+TS | | +0.18 | | +1 |
| H+S +TS | | +13.46[a] | | +2 |
| H+S+A | | +67.78[b] | | +2 |
| A+TS | | +3.05 | | +2 |

H, histology; S, sex; A, age; TS, tumour stage; d.f., degrees of freedom. [a]$P<0.005$; [b]$P<0.001$.

**Table VI** Predicted values of 5-year relative survival calculated from the resultant GLIM model incorporating age and tumour stage

| | *Predicted relative survival* | | |
|---|---|---|---|
| *Age* | *Stage 1* | *Stage 2* | *Stage 3* |
| 0–34 years | 99.8 | 97.6 | 86.3 |
| 35–54 years | 99.5 | 94.7 | 72.0 |
| 55–74 years | 98.5 | 84.3 | 35.7 |

**Figure 4** The 5-year observed relative survival in different stages and age groups.

### Discussion

Our present study includes all verified cases of differentiated thyroid cancer occurring in Norway during the period 1970–79. The registration of clinical data and pathology reports in the Cancer Registry are known to be almost complete. Selection of patients by mode and place of treatment has been a serious problem in most other series. Cases have been collected from different hospitals over long periods of time to obtain appropriate numbers for statistical analysis. This is partly avoided in the present material, gathered from a defined population over a short period of time. Unfortunately the coding of regional lymph node involvement and extrathyroidal extension to the soft tissue has not been consistent or uniform in the period 1970–79. This is the main reason why stage 2 includes both, which could give a rather unhomogeneous patient population in stage 2.

The single factor analysis showed an excellent prognosis of localised tumours after both 5 and 10 years of observation, independent of histological type. The importance of studying the relative survival, thereby obtaining an adjustment for unrelated deaths, is also stressed by this finding. Detailed data on the time and type of tumour recurrences, distant metastases and causes of death would be of great interest.

There were almost no changes in relative survival between 5 and 10 years in stage 1 and 2 tumours. This is somewhat unexpected in view of earlier data (Mazzaferri, 1987) but may be explained by deaths from other causes. It is, however, well known that even well differentiated thyroid cancer may develop metastases up to 15–20 years after first treatment. There are to our knowledge no data indicating an increase in excess deaths with time. When distant metastases are present, the prognosis is poorer, especially among females and in follicular carcinomas. Mortality is furthermore increased between 5 and 10 years after diagnosis, and this was most marked among males.

On the basis of univariate analysis of relative survival, both age and tumour stage had great impact on survival, while sex and histology showed minor variations. Age and stage were the only factors of prognostic importance obtained by the multivariate analysis. Additionally, these two factors were also interrelated. Localised disease presents in the younger patients. Several authors have confirmed the importance of stage (Hannequin *et al.*, 1986; Tubiana *et al.*, 1985; Simpson *et al.*, 1987). One of the advantages of the GLIM package is that it can handle large sets of data with rather small numbers in specific subgroups. We are aware that some of our subgroups (follicular carcinomas in males) fit this category.

It has, however, been debated whether lymph node involvement is of prognostic value. Joensuu *et al.* (1986) found that there was no significant difference in corrected survival between patients with nodal metastases at surgery and those without. Their material is, however, rather small with a total of only 200 patients distributed in three histological types. On the other hand, they claimed that invasion through the capsule was of significant value. Other studies support this, concluding that lymph node involvement is important for local recurrence but not for survival (Tubiana *et al.*, 1985; Hannequin *et al.*, 1986; Simpson *et al.*, 1987). Patients with regional lymph node involvement and extrathyroidal invasion were not analysed separately because of limited data. We have had a preliminary look at our registrated thyroid cancer occurring after 1980, and these data seem to indicate that soft tissue involvement of the neck carries a worse prognosis than nodal metastases.

Our results in the present paper also indicate no difference between stages 1 and 2 below 55 years of age, while those above 55 years had a slightly increased mortality. Finally, stage 3 patients had a clearly increased mortality among all age groups.

There seems to be full agreement among all authors that age is a major prognostic factor (Tubiana *et al.*, 1985; Simpson *et al.*, 1987). It is more uncertain at which age the mortality risk increases. Some authors have observed a decline in relative survival after the age of 40 years (Cady *et al.*, 1985), while Tubiana *et al.* (1985) found this decline appearing some years later. The multifactorial analysis in the present investigation confirms age as a valuable prognostic factor and indicates that the major decline in relative survival appears after the age of 55 years, This is in keeping with Simpson *et al.* (1987), who found more aggressive behaviour in patients beyond the age of 60. The discrepancies may partly be due to different groupings of age. Additionally, differences in stage distribution and histological type may also contribute to such conflicting results. We have shown the impact of age on survival to be clearly dependent on stage, and this may also indicate biological differences between tumours presenting in various stages. The multivariate analysis excludes patients over 75 years. This is done mainly because it is often a problem to give the correct expected survival in older age.

The histopathological typing in this study was based on the WHO classification from 1974 (Hedinger & Sobin, 1974). The slides have not been reviewed, but all reports were examined to adjust the coding. Mixed tumours with both papillary and follicular elements were classified as papillary carcinomas. As stated by Simpson *et al.* (1987), the distinction between papillary and follicular thyroid cancer may be of little more than academic interest. The follicular group was not subdivided with respect to differentiation grade. This may be an explanation of the lack of significant difference in survival between the two histological types. Tubiana *et al.* (1985) found no difference between papillary and well differentiated follicular carcinomas, while follicular tumours of lower differentiation had a worse prognosis. This is confirmed in the EORTC study (Byar *et al.*, 1979) and by Hannequin *et al.* (1986). In view of earlier reports (Saxén *et al.*, 1978), some follicular adenomas may also have been included, but these would only mask a difference of survival in stage 1 tumours. Additionally some papillary carcinomas may be incorrectly diagnosed as follicular carcinomas.

Sex as prognostic factor is also debated, although some studies claim that females have a better final outcome than males (Simpson & McKinney, 1985). We cannot confirm this finding. In our study, which included 803 females and 252 males, no sex differences persist after the multivariate analysis of patients below 75 years of age. This is partly confirmed in a paper by Simpson *et al.* (1987).

One practical conclusion on the discrepancy between different prognostic studies could be that the prognostic factors may change from one population to another (Hannequin *et al.*, 1986). Thus one should be cautious in applying one set of prognostic factors to other populations. On the other hand, there seems to be full agreement that age and stage are independent and important prognostic factors. Studies on cellular DNA content have shown an increasing aneuploidy with age in both benign and malignant thyroid tumours (Joensuu *et al.*, 1986). This may be one explanation of the great prognostic importance of age. It therefore seems to be a clear biological difference between subgroups of thyroid carcinomas. Further studies on cause-specific mortality and tumour recurrences as well as extensive morphological studies are now in progress in Norway.

The Norwegian Thyroid Cancer Project wish to thank The Norwegian Cancer Society for grants and financial support.

### References

BYAR, D.P. *et al.* (1979). A prognostic index for thyroid carcinoma. A study of the EORTC Thyroid Cancer Cooperative Group. *Eur. J. Cancer*, **15**, 1033.

CADY, B., ROSSI, R., SILVERMAN, M. & WOOL, M. (1985). Further evidence of the validity of risk group definition in differentiated thyroid carcinoma. *Surgery*, **6**, 1171.

CANCER REGISTRY OF NORWAY (1975). *Survival of Cancer Patients. Cases Diagnosed in Norway 1953–1967.*

CANCER REGISTRY OF NORWAY (1982). *Trends in Cancer Incidence in Norway 1955–1978.*

GLATTRE, E. *et al.* (1989). Prediagnostic serum selenium in a case-control study of thyroid cancer. *Int. J. Epidemiol.* (in the press).

HAKULINEN, T. & TENKANEN, L. (1987). Regression analysis of relative survival rates. *Appl. Stat.*, **3**, 309.

HANNEQUIN, P., LIEHN, J.C. & DELISLE, M.J. (1986). Multifactorial analysis of survival in thyroid cancer. *Cancer*, **58**, 1749.

HEDINGER, C. & SOBIN, L.H. (1974). *Histological Typing of Thyroid Tumours.* WHO: Geneva.

JOENSUU, H., KLEMI, P., EEROLA, E. & TUOMINEN, J. (1986). Influence of cellular DNA content on survival in differentiated thyroid cancer. *Cancer*, **58**, 2462.

MAZZAFERRI, E.L. (1987). Papillary thyroid carcinoma: Factors influencing prognosis and current therapy. *Semin. Oncol.*, **3**, 315.

SAXÉN, E., FRANSSILA, K., BJARNASON, O., NORMANN, T. & RINGERTZ, N. (1978). Observer variation in histologic classification of thyroid cancer. *Acta Pathol. Microbiol. Scand.* (*A*), **86**, 483.

SIMPSON, W.J. & McKINNEY, S.E. (1985). Canadian survey of thyroid cancer. *Can. Med. Assoc. J.*, **8**, 925.

SIMPSON, W.J., McKINNEY, S.E., CARRUTHERS, J.S., GOSPODAROWICZ, M.K., SUTCLIFFE, S.B. & PANZARELLA, T. (1987). Papillary and follicular thyroid cancer. Prognostic factors in 1,578 patients. *Am. J. Med.*, **83**, 479.

TENNVALL, J., BIÖRKLUND, A., MÖLLER, T., RANSTAM, J. & AKERMAN, M. (1985). Prognostic factors of papillary, follicular and medullary carcinomas of the thyroid gland. *Acta Radiol. Oncol.*, **24**, 17.

THORESEN, S.Ø., GLATTRE, E. & JOHANSEN, A. (1986). Incidence of thyroid cancer in Norway 1970–79. Geographical distribution of histological types. *Tidsskr. Nor. Lægeforen*, **31**, 2612.

THORESEN, S.Ø., MYKING, O., GLATTRE, E., ROOTWELT, K., ANDERSEN, A. & FOSS, O.P. (1988). Serum thyroglobulin as preclinical tumour marker in subgroups of thyroid cancer. *Br. J. Cancer*, **57**, 105.

TUBIANA, M. *et al.* (1985). Long-term results and prognostic factors in patients with differentiated thyroid carcinoma. *Cancer*, **55**, 794.